# Above the Epitranscriptome: RNA Modifications and Stem Cell Identity

**DOI:** 10.3390/genes9070329

**Published:** 2018-06-28

**Authors:** Francesco Morena, Chiara Argentati, Martina Bazzucchi, Carla Emiliani, Sabata Martino

**Affiliations:** 1 Department of Chemistry, Biology and Biotechnologies, University of Perugia, 06126 Perugia, Italy; effemorena@gmail.com (F.M.); chiara.argentati89@gmail.com (C.A.); marti89.b@libero.it (M.B.); carla.emiliani@unipg.it (C.E.); 2 CEMIN, Center of Excellence of Nanostructured Innovative Materials, University of Perugia, 06126 Perugia, Italy

**Keywords:** N6-methyladenosine, N1-methyladenosine, 5-methylcytosine, writers, readers, erasers proteins, epigenetics, mitochondrial transfer RNA, mitochondrial ribosomal RNA, stem cells self-renewal and differentiation, cancer stem cells, naïve and primed stem cells, bioinformatics predictive tools

## Abstract

Sequence databases and transcriptome-wide mapping have revealed different reversible and dynamic chemical modifications of the nitrogen bases of RNA molecules. Modifications occur in coding RNAs and noncoding-RNAs post-transcriptionally and they can influence the RNA structure, metabolism, and function. The result is the expansion of the variety of the transcriptome. In fact, depending on the type of modification, RNA molecules enter into a specific program exerting the role of the player or/and the target in biological and pathological processes. Many research groups are exploring the role of RNA modifications (alias epitranscriptome) in cell proliferation, survival, and in more specialized activities. More recently, the role of RNA modifications has been also explored in stem cell biology. Our understanding in this context is still in its infancy. Available evidence addresses the role of RNA modifications in self-renewal, commitment, and differentiation processes of stem cells. In this review, we will focus on five epitranscriptomic marks: N6-methyladenosine, N1-methyladenosine, 5-methylcytosine, Pseudouridine (Ψ) and Adenosine-to-Inosine editing. We will provide insights into the function and the distribution of these chemical modifications in coding RNAs and noncoding-RNAs. Mainly, we will emphasize the role of epitranscriptomic mechanisms in the biology of naïve, primed, embryonic, adult, and cancer stem cells.

## 1. Epigenetics and Epitranscriptomics

Current knowledge highlights epigenetics as the extra level of the genetic code [1,2,3,4,5]. Epigenetic mechanisms orchestrate molecular events that govern changes in gene expression without changes in the DNA sequence. Epigenetic mechanisms include remodeling of chromatin conformation, methylation and demethylation of DNA, post-translational modification of histones, and the activity of many regulatory proteins [1,2,3,4,5]. The overall events set a gene expression signature that is maintained stable through many cell divisions and is reprogrammed under the effects of specific signals, such as development [6,7,8,9,10,11] and tissue specification [7,8,9,12,13]. Thenceforth, alterations of one of above epigenetic mechanisms are often associated with several diseases, such as cancer and the immunodeficiency–centromeric instability–facial anomalies syndrome (ICF) [14,15].

Recent new knowledge of RNA allows associating the functions of the different RNA classes with the post-transcriptional chemical modifications in the RNAs structure [16,17,18,19] and with the networks of competition between RNA molecules [19,20]. Overall, the interplays are called epitranscriptomics and, definitely, RNA molecules are one of the epigenetic players [16,17,18].

Epitranscriptomic modifications target all types of RNAs and they enclose chemical changes in more than 140 nucleotides [21,22,23,24]. Studies are ongoing to map RNA modifications, to elucidate the biological functions of such modifications and to identify the molecules involved in each modification [1,16,17,25,26,27,28,29,30,31,32].

In this review, we describe the most frequents five RNA modifications, namely N6-methyladenosine, N1-methyladenosine, 5-methylcytosine, Pseudouridine (Ψ) and Adenosine-to-Inosine (A-to-I) editing, together with selected enzymes. We highlight the relevance of bioinformatics tools in the identification of the above-mentioned modifications. Finally, we give insight into the molecular effects of such RNA modifications in stem cell biology.

## 2. RNA Modifications

### 2.1. State of the Art

Biotechnological progress has boosted the understanding of the intricate scenario of modifications in RNA molecules [33,34,35]. Next-generation sequencing and mass spectrometry technologies have revealed widespread messenger RNA (mRNA) modifications together with their effects on mammalian transcriptome [33,34,35]. Many proteins introducing, deleting, or recognizing specific modifications have also been identified and, according to epigenetic language, they have been called as writers, erasers, and readers, respectively [34,35].

The first RNA modification has been identified almost 60 years ago in yeasts [36]. After that, more than 100 modifications in ribonucleotides have been described [36]. These included either common chemical modifications in mRNAs and in noncoding-RNAs or distinct modifications in each type of RNA [36]. The bioinformatics approach is helpful to provide comprehensive databases (e.g., MODOMICS, [36]) with information about the chemical modifications in RNAs and with the biological effects of such modifications.

In this chapter, are reported the latest findings describing the chemical modifications influencing the structure and the function of the different RNA classes: coding RNAs and noncoding-RNAs. Modifications are designed based on the type of chemical changes: N6-methyladenosine (m^6^A) [37,38,39,40]; Pseudouridine (Ψ), 5-methylcytosine (m^5^C) [41]; 5-hydroxymethylcytidine (hm^5^C) [42,43,44], N1-methyladenosine (m^1^A) [45,46], and A-to-I editing [47,48,49]. The mechanism of each modification is described in the mRNA section, as this is the first in this review.

### 2.2. Coding RNA Modifications: The Messenger RNA

#### 2.2.1. Canonical Modifications in mRNA

**Figure 1:**
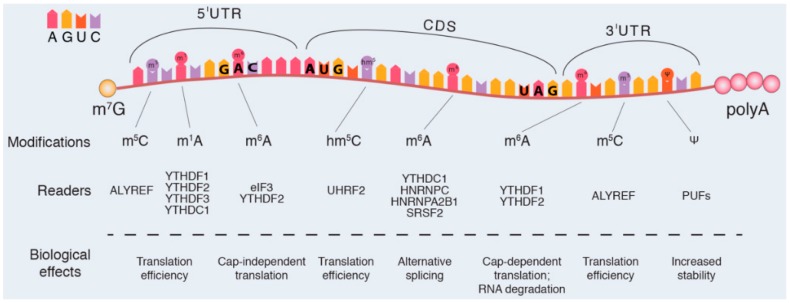
The figure illustrates the possible chemical modifications at the different messenger RNA (mRNA) nitrogen bases. The preferential location of each mark within the mRNA sequence and the reader proteins are also shown (see the text for details). N6-methyladenosine: m^6^A; Pseudouridine: Ψ; 5-methylcytosine: m^5^C; 5-hydroxymethylcytidine: hm^5^C; N1-methyladenosine: m^1^A.

The early canonical modifications in mRNA described in eukaryotes and in some viruses refer to specific changes at the 5′-end and at the 3′-end of this molecule [50,51,52] (Figure 1).

The first modification, called as cap-0, comprises the addition of an N7-methylguanosine (m^7^G) via a 5′–5′ triphosphate bond to the 5′-terminal nucleotide of the pre-mRNA [53,54]. Alternatively, in small nuclear RNAs, the guanosine is modified by the addition of 2 methyl groups at the N2 position. The three-methylguanosine cap allows the interaction with U1, U2, U4, and U5 ribonucleoproteins (snRNPs) and it is required for pre-mRNA splicing and pre-ribosomal RNA (rRNA) processing if the modification interacts with U3 and U8 snRNPs [55].

Further modifications at 5′-end mRNA (called as cap-1 and cap-2) consist of a ribose 2′-*O*-methylation on the first and second transcribed nucleoside [56]. Cap-1 and Cap-2 are critical for RNA processing, translation, and stability [50,51].

The second main mRNA modification arises at the 3′-end and it is called as poly(A)-tail. It comprises at least 150–200 adenylate residues and a conserved AAUAAA sequence bordered by less conserved sequences (e.g., downstream elements composed of U-rich sequences). The poly(A) tail guarantees mRNA stability through the export out of the nucleus and during the translation process [51,52,57].

In higher eukaryotes, a third well-documented modification occurs. It relates to the intron excision from pre-mRNAs through the splicing mechanism achieved by the splicing machinery comprising of U1, U2, U4, U5, and U6 snRNPs, in addition to other non-snRNP proteins [52,58,59]. Thanks to the splicing process, pre-mRNA precursors enter into the post-transcriptional regulation of gene expression and give rise the landscape of proteome in the cells [60].

#### 2.2.2. Non-Canonical mRNA Modifications

**Figure 2:**
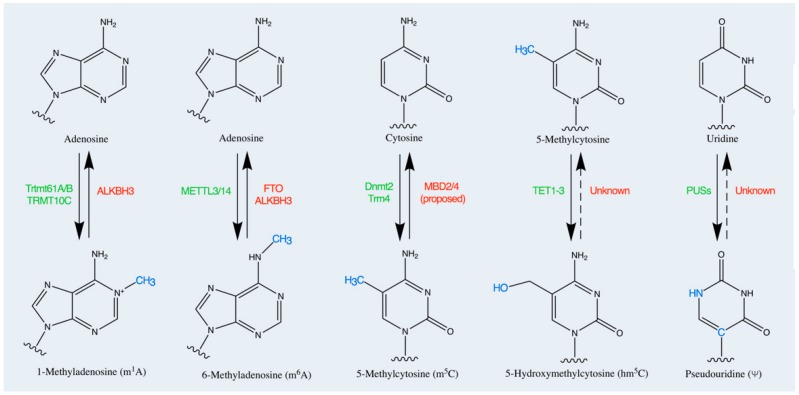
The figure illustrates the chemical modifications at the Adenosine, Cytosine, and Uridine nitrogen bases. In green are the enzymes catalyzing the reaction of addition of a moiety group (writers). In red are the enzymes catalyzing the reaction of deletion of the modification (erasers). The modification sites are colored in blue. The abbreviations of the modified nucleosides are shown in parentheses.

In addition to the above mentioned modifications, other nucleosides carrying chemical modifications at the nitrogen bases, have been discovered in mature mRNAs, in cytoplasmatic mRNA (Figure 1 and Figure 2) [33,47,50,61,62] and in mitochondrial mRNA (mt-mRNA) [63,64].

The molecular characteristics of each modification, together with the writers, readers, and erasers, are detailed below.

**N6-methyladenosine** consists of the addition of a methyl group at the nitrogen-6 position of adenosine base in the sequence GAC [18] (Figure 1). The m^6^A modification in 5′-untranslated regions (5′-UTRs) was the first internal mRNA modification uncovered, several decades ago [18,33,43,44,45,65]. At the same time, the methyltransferases involved in the methylation process (writers) [66,67] and the proteins specifically recognizing the m^6^A decoy (readers) [18,68] were identified (Figure 1 and Figure 2).

METTL3 methyltransferase (alias MT-A70), was the first enzyme identified as part of a large multimeric protein complex involved in the mRNA methylation, in 1997 [66]. Now, it is known that this enzyme (belongs to the DNA m^6^A methyltransferases subfamily) contains the sequence D/N/S/H]PP[Y/F/W, highly conserved among yeasts, plants, Drosophila, and mammals [69]. Recent studies found the METTL14 as another component of the writer complex. METTL3 serves as a catalytic subunit while METTL14 functions as a stabilizer for the reaction [70]. However, both proteins expose a binding site for RNA in the consensus sequence RRACH (R is a purine, A corresponds to the m^6^A modification and H is a non-guanine base [69]).

The reader proteins specifically recognize the m^6^A decoy, due to the presence of YTH sequence, (the specific m^6^A -reader domain [18,68]) and control the mRNA homeostasis. For instance, the reader YTHDF2 regulates the mRNA stability, the YTHDC1 controls the splicing of mRNA, whereas the YTHDF1 and YTHDF3 supervise the translation process [71,72,73] (Figure 2).

The fat mass obesity-associated protein (FTO) [22,23] and AlkB Homolog 5 RNA Demethylase (ALKBH5) [74,75], two members of the Fe(II)-dependent and 2-oxoglutarate-dependent oxygenase superfamily, are involved in the removal of m^6^A modification in RNAs in vitro and in vivo [22,23] (Figure 2). FTO binds to the m^6^A moiety in mRNAs and converts m^6^A to N6-hydroxymethyladenosine (hm^6^A) and N6-formyladenosine (f6A) in a step-wise manner. Studies are in progress to uncover additional proteins that might be involved in promoting the FTO-mediated demethylation of m^6^A [22,23].

Noteworthy, m^6^A has also been observed in the 3′-UTR region of mRNA. This modification, together with the readers YTHDF1 and YTHDF2, controls the mRNA stability during the stem cell differentiation or the circadian clock control [18,76]. Additionally, YTHDF2 binds to m^6^A in the 5′-UTR of stress-responsive mRNAs, prevents the FTO-mediated demethylation through a direct bond via eIF3 complex (eukaryotic translation initiation factor 3) and permits a rapid cap-independent translation of these mRNAs [77,78,79,80].

m^6^A demethylation on NANOG mRNA was observed in breast cancer stem cells in response to hypoxia. In these cells, the hypoxia-inducible factor activates the demethylase ALKBH5 and thereby the removal of m^6^A [81]. Indeed, the m^6^A methylation of mRNA seems a mechanism that cells employ in response to various stresses, such as deprivation of nutrients, deficit of oxygen, exposure to extreme temperatures or toxins [80,82]. Thus, budding yeasts respond to nitrogen starvation entering meiosis by the m^6^A methylation of key transcripts [80].

**N1-methyladenosine.** The N1-methyladenosine modification, uncovered in all classes of RNAs several decades ago [83], has been revealed in mRNA signature in eukaryotic cells from yeast and mammals (Figure 1 and Figure 2). The m^1^A modification consists of the addition of a methyl group at the nitrogen-1 position of adenosine [83]. m^1^A is copious in GC-rich regions of the 5′-UTRs regions but it’s about ten times less abundant than m^6^A. The m^1^A modification is specifically recognized by YTHDF1, YTHDF2, YTHDF3, and YTHDC1 reader proteins [84]. The role of m^1^A modification is under elucidation, however, first evidence suggests that the addition of m^1^A to mRNA is critical for the initiation of translation process through the binding with unknown readers [45] (Figure 2).

**5-Methylcytosine**. Currently, the potential roles of m^5^C in mRNA remain elusive. Methylation at position 5′ of cytosine increases the hydrophobicity of mRNA and probably increases base stacking but has a minimal effect on base pairing [26] (Figure 1 and Figure 2). The m^5^C modification is specifically recognized by the reader enzyme ALYREF [85]. m^5^C sites seem to be widespread randomly in coding regions, although an enrichment has been also observed in the 5′-UTR and the 3′-UTR regions (Figure 2). For instance, the methylation of p16 mRNA by NSUN2 (an RNA m^5^C methyltransferase) increases the stability of the p16 transcript and prevents the degradation [42,86].

**5-Hydroxymethylcytosine** modification is highest in exon sequences and increases the efficiency of translation (Figure 1 and Figure 2). The exact mechanism is still unknown but it is likely that the m^5^C might be subjected to oxidative processing forming hm^5^C [77,87]. In *Drosophila melanogaster*, levels of hm^5^C are highest in the brain, suggesting a potential role in brain development [77,87].

**Pseudouridine** modification in the mRNA is a recent finding, contrasting with evidence showing that pseudouridylation is the most abundant modification in the noncoding-RNAs [88,89]. The Ψ modification consists of the isomerization of uridine in which the uracil is attached via a carbon–carbon bond instead of a nitrogen–carbon glycosidic bond [88,89]. Ψ reader proteins have not yet been identified, although the PUFs family (Pumilio family proteins) seems a good candidate [90]. The Ψ in mRNA 3′-UTRs has been shown to increase the stability of modified transcripts during heat shock processes [88,89] (Figure 1 and Figure 2). It was also suggested that mRNA pseudouridylation could alter the efficiency of the translation initiation and it could influence ribosome pausing and RNA localization [88,89].

**Adenosine-to-inosine editing**. Inosine modification, also referred to as Adenosine-to-Inosine (A-to-I) editing, consisting of different base-pairing properties, has been described in mRNA transcripts [77,91]. Inosine pairs were also observed in the combination Inosine-to-Cytosine (I-to-C), that is more stable [77,91]. This modification is critical for the editing of mRNA and therefore for the fate of mRNA [77,91,92].

#### 2.2.3. Mitochondrial mRNA

Modifications in the mRNAs of mammalian mitochondrial have revealed recently by transcriptome-wide analyses [93]. It is suggested that mitochondrial mRNA (mt-mRNA) modifications are necessary for facilitating the mitochondrial gene expression machinery, due to the minimal set of transfer RNA (tRNAs) and to the non-conventional genetic code [63,64,93,94]. For instance, the methyltransferase TRMT10C adds m^1^A in a single site in the NADH-ubiquinone oxidoreductase chain 5 mt-mRNA, leading its post-translation modification [95]. In yeasts the presence of the m^6^A in key transcripts, by mitochondrial m^6^A methyltransferase IME4 activity, is necessary for meiosis and sporulation in yeasts [96]. The C-to-U editing in mt-mRNAs seems a characteristic of transcripts in the plant model moss *Physcomitrella* [97].

### 2.3. Noncoding-RNA Modifications

To offer a comprehensive landscape of the epitranscriptome, here, we summarized the post-translation modifications in noncoding-RNAs:tRNAs, rRNAs, and regulatory RNAs (such as long noncoding RNAs [lncRNAs], microRNA [miRNA], and circleRNA [circRNA]) [40,75,98,99]. The five main modifications, m^6^A, m^1^A, m^5^C, and Ψ and A-to-I editing, have been documented in noncoding-RNAs.

Apart from tRNA and rRNA, the knowledge on the modifications occurring in other types of noncoding-RNAs and their effects on cellular functions is still restricted to a few studies.

#### 2.3.1. Transfer RNA

The presence of chemical modifications in numerous nucleotides in mature tRNA is largely studied. The role of modifications in the function of tRNA has been demonstrated (see Table 1 for more details and for review [100,101,102]). Different modifications have been identified in the tRNA anticodon loop (e.g., inosine, queuosine, 5-methylcytosine, 5-methoxycarbonylmethyl-2-thiouridine, threonyl-carbamoyl-adenosine and wybutosine), as required for the interaction of tRNA and mRNA within the ribosome [74,92,103,104,105,106,107,108,109]. Other modifications have been found associated with stability, the localization, ribosome binding, and translational dynamic processes [100,110]. For instance, A-to-I editing at wobble positions was found in at least eight human tRNA samples and it was correlated with the expansion of the base pairing capability from A34-U to I34-U/I34-C enhancing the codon–anticodon interactions in the ribosome [110]. The uracil methylation at position 5 (m^5^U) by the enzyme hTRMT9 has been also described in tRNA and it was associated with an increase in decoding activity [111,112]. More recently, the presence of the m^1^A modification at nucleotides 9, 14, 22, 57, and 58 was associated with increased tRNA stability and with the correct tRNA folding [113]. Other modifications in tRNA nucleotides (such as N1-methylguanosine, N6-threonylcarbamoyladenosine, N6-isopentenyladenosine) have been involved in preventing frameshifting or in helping the tRNA on the ribosome accommodation during the translational elongation process [100,114].

The tRNA methyltransferase family is involved in these reactions. Thus, TrmL is the writer that catalyzes the transfer of a methyl group to the 2′-hydroxyl group of the pyrimidines at the wobble position 34 in two tRNA isoacceptors [115,116,117]. However, MS analysis has revealed that METTL2 is the enzyme catalyzing 3-methylcytosine (m^3^C) methylation in total tRNA and that METTL6 is the enzyme catalyzing m^3^C in seryl-tRNA [118].

Recent studies have suggested a link between defects in tRNA modifications and neurological disorders, such as Amyotrophic Lateral Sclerosis (ALS) [119]. It was shown that ELP3, a subunit of the elongator complex modifying wobble uridines in tRNA, attenuated the axonopathy of a mutant SOD1 of ALS zebrafish model [120].

**Mitochondrial tRNA (mt-tRNA)**. The role of epitranscriptomic modifications in mt-tRNA have been extensively described [101,121,122]. For example, the eraser ALKBH1 catalyzes 5-hydroxymethyl-2′-*O*-methylcytidine and 5-formyl-2′-*O*-methylcytidine modifications at the first position of the anticodon of mitochondrial tRNAMet. These modifications are essential for the translation of the non-universal codon AUA in mammalian mitochondria [114]. Further, the modification at the wobble position of selected mt-tRNAs of the uridine base in taurinomethyluridine is necessary to avoid ribosome stalling at certain AAG (lysine) and UUG (leucine) codons [123]. The reduction of mt-tRNA m^5^C methylation and formylation impairs the mitochondrial translation and respiration processes [124].

#### 2.3.2. Ribosomal RNA

rRNA modification has been revealed in several nucleotide residues [117,125,126]. During the biogenesis of ribosomes, a large fraction of rRNA nucleotides (approximately 2% on human ribosomes) is specifically modified, either by writer enzymes or by antisense small nucleolar RNAs [117,125,126]. The most abundant modification is represented by 2′-*O*-methylation (2′-*O*-Me) or Uridine-to-Pseudouridine isomerization. Methylations of mature rRNA may be achieved through the action of specific methyltransferases, which are responsible for the modification of one or sometimes two specific nucleotides generally during ribosome assembly [127]. Epitranscriptomic rRNA modifications play an important role in the rRNA catalytic activity, folding, and stability and it is likely to modulate the antibiotics action. Interestingly, the presence of unexpected rRNA modification sites and the absence of the predicted 2′-*O*-Me sites have revealed that rRNA modifications differ among human cell types, as well as between healthy and cancer cells, such as hepatocellular carcinoma and breast cancer [128]. An example of the isomerization of Uridine to Ψ is the modification described in 23S-rRNA, by the pseudouridine sintase RluE, and required for RNA–protein interaction and Ψ formation [129]. More recently, the m^1^A modification has been documented in yeast 25S-rRNA and mammalian 28S-rRNA, respectively [130]. In particular, the m^1^A in 28S-rRNA was associated with the control of the translation process during embryogenesis and erythropoiesis. It was shown that KO of the nucleomethylin caused the defective methylation of rRNA and the consequent failure of ribosome formation [131].

**Mitochondrial rRNA (mt-rRNA)**. Epitranscriptomic modifications in mt-rRNA are documented in all type of rRNAs [132]. The 2′-*O*-methyl modification, at position U1369 in the human 16S-mt-rRNA and at position U279 in the yeast 21S-mt-rRNA, is necessary for the correct respiration machinery [126]. The pseudouridylation of a single residue in the 16S-mt-rRNA is required for the oxidative phosphorylation complex assembly as demonstrated by the depletion of the Pseudouridine synthases, TRUB2, RPUSD3, and RPUSD4 through the CRISPR/Cas9 system [133]. The m^5^C modification at position 911 of cytosine in 12S rRNA by the methyltransferase NSUN4 activity, is critical to control the final step in ribosome biogenesis and to guarantee that only the mature major and minor ribosomal subunits are assembled [134].

#### 2.3.3. Long Noncoding-RNA

m^6^A, m^5^C, A-to-I editing, and Ψ modifications have been documented in mature lncRNAs (Table 1) [104,135]. For instance, a segment with 78 m^6^A residues was shown critical for regulating the lncRNA X-inactive specific transcript (XIST) activity in silencing genes in the X chromosome [93]. The m^6^A formation in XIST is mediated by the RNA-binding motif protein 15 (RBM15), which binds the m^6^A methylation complex recruiting it to specific sites in the lncRNA [93]. m^6^A modification is also implicated in the destabilization of the lncRNA hairpin stem, allowing the access of heterogeneous nuclear ribonucleoproteins C1/C2 (HNRNPC) to a U-rich tract situated on the hairpin arm opposite of the m^6^A in Metastasis-Associated Lung Adenocarcinoma Transcript 1 (MALAT1) lncRNA [72].

#### 2.3.4. microRNAs

Chemical modifications have been uncovered in mature miRNAs (Table 1). Methylation of 2′-*O*-methyl group at 3′-end of miRNA by the HEN1 methyltransferase is necessary for protecting these small RNAs from poly-uridylation and degradation [136]. A-to-I editing has been demonstrated in miRNAs. In this regard, Shoshan and co-workers correlated A-to-I editing of miR-455-5p with the metastatic activity of melanoma cancer [137].

#### 2.3.5. circRNAs

m^6^A modifications have been revealed in circRNAs (Table 1), combining the computational pipeline AutoCirc and immunoprecipitation assays. A further study demonstrated that the readers YTHDF1 and YTHDF2 also interact with circRNAs [138].

## 3. Bioinformatics for Epitranscriptomics

To provide an integrative analysis of epitranscriptome-wide landscapes of RNA modifications from high-throughput sequencing, different types of databases have been developed. The availability of large epitranscriptome-wide datasets for m^1^A, m^6^A, m^5^C, and pseudouridine have stimulated the creation of several databases, periodically updated.

### 3.1. Databases for RNA Modifications

Here are reported the characteristics of the most renowned RNA modifications databases: The RNA Modification Database (RNAMDB) [139], MODOMICS—a database of RNA modifications pathways [78], MeT-DB—a database of transcriptome methylation in mammalian cells [140], and a database dedicated to RNA modifications in normal and disease such as RNA Modification Base (RMBase) [141].

**RNAMDB** is online from 1997 [142]. Acting as a reference for updating findings on RNA modifications. RNAMDB reports 13 eukarya; mRNA known post-transcriptional modifications that naturally occurring in mRNAs and 11 eukarya; small nuclear RNA (snRNA) in noncoding-RNA species as miRNAs, snRNAs, and small nucleolar (snoRNAs). Presently, the database for each entry provides the information on chemical structures, common name, symbols, molecular weight, Chemical Abstracts index name, and registry numbers [139]. The RNAMDB portal also hosts a number of tools of great benefit in mass spectrometry identification and characterization of natural or modified RNAs. Starting from an RNA sequence, it is possible to calculate the molecular mass, electrospray series, CID fragments, internal fragments, base losses, and fragment digestions [139].

**MODOMICS**. The MODOMICS database represents the reference database of RNA modifications since it provides the most complete information regarding the chemical structures of modified ribonucleosides, the reaction summary, the functionally characterized enzymes involved in modifications, and the biosynthetic pathways of RNA modifications. The current database version contains 163 different modifications that have been identified in RNA molecules and about 340 functionally characterized proteins involved in RNA modifications [78]. MODOMICS also provides information of (i) Liquid chromatography-Mass Spectrometry (LC-MS) data for modified nucleosides; (ii) Simplified molecular-input line-entry system (SMILES) for each modified base with the tridimensional structure (e.g., MDL Molfile, .mol) and its occurrence in Protein Data Base (PDB) structures. All files can be downloaded from their site and are useful in the field of the molecular dynamics and simulation [143,144,145,146].

**MeT-DB**. The knowledge about m^6^A position sites plays an essential role in investigating the mechanisms and functions of this modification.

MeT-DB is a comprehensive database focusing on m^6^A mammalian methyltranscriptome [140]. MeT-DB includes approximately 300.000 m^6^A methylation sites detected in samples from different experimental conditions, in human and mouse cells [140]. Data were obtained from methylated RNA immunoprecipitation sequencing (MeRIP-Seq) analyses [147] and were detected by exomePeak and MACS2 algorithm [148,149,150]. To explore the whole information, MeT-DB provides a genome browser to query and visualize specific m^6^A methylation. MeT-DB also includes in the browser window the data on binding site of microRNA, on splicing factor and on RNA binding proteins for comparison with m^6^A sites and for exploring the potential functions of m^6^A.

**RMBase**. The RMBase is a comprehensive database that integrates epitranscriptome sequencing data for exploring the post-transcriptional modifications of RNAs and their relationships with miRNA, disease-related single-nucleotide polymorphisms (SNPs), and RNA-binding proteins (RBPs). Since its inauguration, RMBase provides a variety of interfaces and graphic visualizations to facilitate analyses of the massive modification sites in normal tissues and cancer cells [151]. Currently, the RMBase v2.0, contains ~5400 m^1^A, ~1,373,000 m^6^A, ~5100 2′-*O*-methylations, ~9600 pseudouridine modifications, ~1000 m^5^C modifications, and ~2800 modifications of other types, identified from high-throughput sequencing data (MeRIP-seq, m^6^A-seq, miCLIP, m^6^A-CLIP, Pseudo-seq, Ψ-seq, CeU-seq, Aza-IP, RiboMeth-seq) [141]. RMBase also includes a motif module providing the visualized logos and position weight matrices (PWMs) of the modification motifs; a modRBP module to study the relationships between RNA modifications and RBPs. RMBase identified thousands of nucleotide modifications within mRNAs, regulatory noncoding-RNAs (e.g., lncRNAs, miRNAs, pseudogenes, circRNAs, snoRNAs, tRNAs), miRNA target sites, and disease-related SNPs.

### 3.2. Bioinformatic Tools for Predicting RNA Modifications

The development of an in silico approach based on support vector machines (SVM) [152,153,154] to accurately predict post-transcriptional modification sites from sequence information, is very helpful for the scientific community [154]. As good complements of experiments, many computational methods have been proposed to predict RNA modification sites in recent years [153,155,156,157,158,159,160,161,162,163,164,165,166,167,168,169]. In Table 2 are summarized the online computational tools (user-friendly web server) currently existing directed at predicting the RNA modification sites.

## 4. Epitranscriptomics and Stem Cells

### 4.1. Stem Cells

Stem cells are the unique cells with the capability to keep the stemness feature (self-renewal property) and to generate differentiate progenies in response to selective signals (pluri/multipotency property). Both activities are the consequence of the asymmetric division of stem cells producing two different daughter cells: one identical to the mother stem cell, and one committed toward a selected cell lineage. In some cases, stem cells divide in a symmetric division and produce two stem cells, both identical to the mother cell [170,171,172].

Naïve and primed stem cells represent the first distinction between the stem cell types [173,174]. Both stem cells originate during the zygotic stage of the mammalian embryo, immediately after the maternal predetermination. This stage, considered the ground zero status for embryogenesis, is a transient window in the preimplantation epiblast where unrestricted stem cells (naïve) are generated and, are then, set to enter into a lineage-commitment process (primed stem cells) [173,175]. This initial difference is extended to stem cells derived from embryos or through reprogramming techniques *ex vivo* [175]. Thus, embryonic stem cells (ESCs) are the stem cells derived by the immortalization of the naïve epiblast [176], while the primed stem cells, are the stem cells derived from post-implantation epiblasts and therefore are termed epiblast stem cells (EpiSCs) [173,175]. The latter cells express the Oct4, Sox2, and Nanog pluripotency genes and differ from ESCs for the differentiation efficiency [173,175].

The natural stem cell types (ESCs, EpiSCs, and adult stem cells, ASCs, [170,177,178]), and those generated in vitro (induced pluripotent stem cells, iPSCs [179,180,181]) have self-renewal properties but differ for the capability to generate differentiated cells (see Table 3 for details). Moreover, ASCs have the capability to replace damaged cells with new healthy substitutes within the adult tissues/organs where they reside or after therapeutic implantation, as this property is maintained in degenerated tissues/organs [17,181,182,183,184,185,186]. Furthermore, the regenerative potential may be enhanced by the combination of the stem cells with gene therapy technology [187,188,189,190,191,192,193,194,195] or by their association with selected biomaterials [174,196,197,198,199,200].

Noteworthy, since the last decade, cancer stem cells (CSCs) have been recognized as the stem cell type causing tumors progression. For these features, CSCs are considered a challenge for cancer therapies [201,202,203]. CSCs have self-renewal and multipotential properties as well as several other characteristics critical for metastatic development (e.g., as motility, invasiveness, and resistance to therapeutics) (Table 3; [201,202,203]).

### 4.2. RNA Modifications and Stem Cells

Stem cells’ functions are orchestrated by networks of molecular cues including both epigenetic (e.g., reorganization of chromatin architecture, DNA methylation, and demethylation, post-translational modifications of histones), and epitranscriptomic signals (e.g., RNA modifications and related proteins). The whole processes determine genes expression signature and, in turn, stem cells’ maintenance and specification lineage processes [17,186,204,205,206,207,208,209,210,211].

In the last chapter, we describe the role of epitranscriptomic modifications on the stem cells’ processes, highlighting the effects of different RNA modifications on stem cell fate.

#### 4.2.1. N6-methyl-adenosine mRNA Modification and Stem Cells

Among RNA modifications, the presence of m^6^A in mRNAs has been explored in several types of stem cells. Key roles in modulating stem cell maintenance, differentiation, reprogramming, and controlling of the development stages in mammals have been revealed [38,212,213,214].

**Naïve vs. primed stem cells**. Recent studies suggest that m^6^A modification of mRNA could be useful for facilitating the distinction between naïve and primed cells (Table 4). This conclusion primarily comes from the study of Geula and collaborators [215]. This group demonstrated that m^6^A mRNA methylation is a molecular switch, acting as a regulator during the resolution of murine naïve pluripotency. The methylation seems critical for the safeguarding and timely downregulation of pluripotency factors, which is required for proper lineage priming and differentiation. This mechanism was confirmed by METTL3–/– knockout that leads to the depletion of m^6^A in mRNAs in the blastocysts. Here, METTL3 ablation retained the normal morphology and expression of pluripotency genes that produced ESCs at the expected ratio. Additionally, METTL3–/– ESCs preserved their naïve pluripotent identity, whereas METTL3 KO ESCs did not support the embryo chimera formation after blastocyst microinjection [215]. More importantly, METTL3 depletion reduced the expression of pluripotency genes, greatly increased the lineage commitment markers and, in turn, lead the tipping off of the balance to differentiation compromising the stability of the primed state [215].

Table 4 summarizes the effect of the different RNA modifications in the naïve, primed, ESCs, ASCs, and CSCs.

These findings were also confirmed by the study of Aguilo and co-workers [216]. It was shown that early inactivation of METTL3 during mouse development blocked the reprogramming of differentiated EpiSCs to ESCs. Zinc finger protein 217 is partially responsible for stabilizing key pluripotency and for reprogramming transcripts by inhibiting their METTL3-mediated methylation, thus promoting the self-renewal of ESCs and the reprogramming of somatic cells [216].

**Embryonic stem cells**. The role of m^6^A methylation of mRNAs in the pluripotency and in the differentiation processes of ESCs is being currently explored [213,214,217,218]. So far, results are yet contrasting since they support the role of m^6^A in opposite processes (Table 4).

Some authors endorsed m^6^A modifications as critical steps for keeping ESCs in a stemness status [213,217,218]. Thus, the knock-down of both METTL3 and METTL14 in mouse ESCs resulted in reduced levels of m^6^A modification and, in turn, in decreased stemness activity and increased differentiation program of stem cells. In particular, the knock-down of METTL3 and METTL14 caused a decrease in the expression of pluripotency genes (e.g., NANOG and SOX2) and an increase in the expression of developmental regulators, although in part mediated by the human antigen R and several microRNAs (e.g., let-7a-3) [67,217,218]. Supporting this study, Wang and collaborators suggested that m^6^A modification enhances the self-renewal activity in embryonic neural stem cells. In METTL14 knockout mice, embryonic neural stem cells displayed high decreased proliferation and premature differentiation and, in turn, less number of late-born neurons during cortical neurogenesis [217]. Furthermore, germ cell-specific METTL3 knockout mice impaired the initiation of meiosis and, in turn, male fertility, and spermatogenesis [219].

TGFβ signaling supports these findings. In fact, SMAD2 and SMAD3, through the interaction with the METTL3-METTL14-WTAP complex, promote binding of the m^6^A methyltransferase complex to transcripts involved in early cell fate decisions. This mechanism destabilizes specific SMAD2/3 transcriptional targets, including the pluripotency factor gene NANOG [220].

Challenging the above reported conclusions, other authors suggested the need of m^6^A modifications for the transition of ESCs to differentiation stage [215,221]. Depletion of m^6^A by METTL14 knockout in embryonic mice brains has been shown to prolong the cell cycle of radial glial cells and to extend the cortical neurogenesis into postnatal stages. m^6^A sequencing of embryonic mice cortex revealed enrichment of mRNAs related to transcription factors involved in neurogenesis, cell cycle, and neuronal differentiation [222]. Convergent in vitro and in vivo studies revealed that gene silencing or depleting of murine and human METTL3 caused the removal of m^6^A in target genes, sustained the expression of NANOG, and blocked the activation of ESC commitment program toward the diverse differentiation lineages. Therefore, m^6^A has been proposed as a mark of a transcriptome required for stem cells to enter in the differentiation status [221,223].

**Adult stem cells**. Many research groups have investigated the effect of m^6^A mRNA modification in adult stem cells.

An indirect correlation of the presence of m^6^A modifications in mRNAs and in differentiation processes was observed by Vu et al. [224]. It was shown that silencing of METTL3 by interference RNA transfection induced the differentiation of human hematopoietic stem/progenitor cells (hHSPCs), whereas the overexpression of wild-type METTL3 caused inhibition of stem cell differentiation [224].

Additional evidence comes from the need of m^6^A methylation in the 5′-UTR of MyoD mRNA for correct expression of MyoD protein in proliferative myoblasts. This transcription factor is necessary for myogenic stem/progenitor cells to retain their skeletal muscle differentiation potential [225]. Using MeRIP-seq [226] to profile mRNA modifications in zebrafish embryos at 28 h post fertilization, Zhang and coworkers studied the role of m^6^A mRNA methylation in the stem cell fate [227]. Generating METTL3-deficient zebrafish embryos, they observed that the decreased levels of m^6^A hampered the development of hematopoietic stem cell. In particular, they showed that the reader YTHDF2 mediates the degradation of NOTCH1a RNA [228]. These findings were also confirmed in knockdown METTL3 mice [213,227,229].

**Cancer stem cells**. The relevance of m^6^A modification in mRNA functions in stem cell biology is highlighted by the correlation of abnormal m^6^A profile in malignant cells (Table 4). These findings include also the expression of reader, writer, and eraser proteins.

For instance, Weng and coauthors observed the increase of the expression of m^6^A methyltransferase METTL14 in healthy HSPCs and acute myeloid leukemia (AML) cells carrying the t(11q23), t(15;17), or t(8;21) mutations, and its downregulation during myeloid differentiation [230]. The parallel increase of m^6^A modifications and METTL3 expression was also demonstrated in c-MYC, BCL2, and PTEN mRNAs in the human AML MOLM-13 cells line [224]. Furthermore, silencing of METTL14 has been found to promote terminal myeloid differentiation of normal HSPCs and AML cells and to inhibit AML cells survival and proliferation. Like METTL3, METTL14 has been demonstrated to exert its oncogenic activity by regulating the m^6^A levels in MYB and MYC mRNAs while the methyltransferase itself is inhibited by SPI1. Thus, the SPI1-METTL14-MYB/MYC signaling is suggested as a basic mechanism for myelopoiesis and leukemogenesis, whereas the levels of METTL14 and m^6^A modification are critical in normal and malignant hematopoiesis [230]. In this context, the protein CEBPZ (CCAAT/Enhancer Binding Protein Zeta) has been identified as the mark required to recruit of METTL3 to chromatin and to maintain the leukemic state [231].

The knockdown of METTL3 or METTL14 was also found to promote the growth and self-renewal of human glioblastoma stem cells (GSCs) and the progression of tumorigenesis. Additionally, the overexpression of METTL3 and the inhibition of the RNA demethylase FTO was shown to arrest GSC growth and the self-renewal processes [232].

Validating these data, Zheng and collaborators showed high levels of the m^6^A demethylase ALKBH5 in human GSCs [233]. Moreover, the silencing of ALKBH5 suppresses the proliferation of GSCs in vitro through the alteration of m^6^A methylation of some mRNA target of ALKBH5. In particular, ALKBH5 demethylates FOXM1 mRNA and controls FOXM1 protein expression by the long noncoding-RNA FOXM1-AS that led to blockage of tumorigenesis of GSC [234].

Alteration of m^6^A methylation, due to the dysfunction of METTL3, has been also associated with the progression of human lung cancer and foremost with growth, survival and invasion of human lung cancer cells. It has been demonstrated that in human cancer cells, METTL3 promotes the translation of certain mRNAs, including the EGFR and the TAZ protein [235].

Of note, deregulation of epitranscriptomic events in cancer biogenesis and progression comprises also adenosine-to-inosine double-stranded RNA editing (e.g., pri-microRNA let-7), by the activity of the enzyme adenosine deaminase [236].

In MDA-MB-231 breast cancer cells, the knockdown of the eraser ALKBH5 caused the demethylation of m^6^A in NANOG mRNA and significantly decreased metastasis from breast to lungs in immunodeficient mice. A similar mechanism could be induced by the exposure of breast cancer cells to hypoxia that specifically induces ZNF217-dependent inhibition of m^6^A methylation [81,237].

#### 4.2.2. 5-methylcytosine and 5-hydroxymethylcytosine Modifications and Stem Cells

Knowledge about the prevalence and transcriptome-wide distribution of m^5^C in stem cells is still limited. Nevertheless, studies in different cell types, tissues, and organisms correlated the m^5^C and the hm^5^C modifications with key biological pathways [238] (Table 4). For example, the activity of MISU/NSUN2 (the writer of m^5^C methylation) was shown to be essential in controlling of the expression of critical transcripts critical in the balance of epidermis stem cell self-renewal and differentiation processes [238,239]. Advances could come from epitranscriptome profile studies. A global analysis of m^5^C in whole and nuclear poly(A) RNA of mice embryonic stem cells and brain was conducted by Amort and collaborators [240]. In particular, a marked accumulation of m^5^C sites close to of the translational start codon, a reduction in coding sequences, and an enrichment in the 3′-UTR [240] were observed.

In ESCs, the m^5^C of NSUN3 mt-tRNA is associated with the stem cell fate regulation. Moreover, NSUN3 inactivation attenuated induction of mitochondrial reactive oxygen species (ROS) upon stress, which may affect gene expression programs upon differentiation. These findings indicated NSUN3 as a central regulator of stem cell fate and also provide a model system to study the correlation of mitochondrial function with stem cell pluripotency and differentiation [124].

#### 4.2.3. Pseudouridine Modification and Stem Cells

As aforementioned, pseudouridine modification has been demonstrated in whole RNA types (Table 4). Nevertheless, evidence correlating this modification and stem cell biology are restricted to few studies. Most of them highlight the correlation of levels of expression of pseudouridylation writers with the control of stem cell differentiation, as well as, with pathways essential for normal development [211,241,242]. A recent study showed that during the first stage of embryogenesis, the pseudouridylation of tRNA is directly involved in the stem cell commitment [242,243]. Guzzi and co-authors identified the PUS7 as the writer of above modifications and found their localization into the tRNA-derived fragment, the region involved in the translation initiation complex [243].

## 5. Concluding Remarks

The discovery of RNA modifications opened a new frontier in the understanding of the biological processes. Identification of over 100 modifications in coding and noncoding-RNA nucleotides has revealed a variety of interplays involving RNA molecules directly or RNA molecules that get over the competition between mRNAs and noncoding-RNAs. Our knowledge in this field is in the early days. However, it can be predicted that elucidation of the epitranscriptome regulatory mechanisms could help the understanding of how gene expression is finely tuned and regulated across development and in tissues.

Future studies aimed at deciphering the epitranscriptome in stem cells could shed light on the mechanisms of stem cell self-renewal and stem cell differentiation. In particular, the combination of bioinformatic predictive tools together with single-cell epitranscriptomic sequencing could provide insights into the molecular differences underlying naïve and primed stem cells. The same approach might be advantageous for understanding the mechanisms controlling the commitment and the lineage specification of stem cells.

Furthermore, better knowledge of epitranscriptomic mechanisms could help the development of inhibitors to target writers, readers, and erasers, and thereby to explore new routes for controlling gene expression in stem cells in physiology and pathology. Such discoveries might aid the design of innovative therapeutic strategies for complex diseases, including cancer.

## Figures and Tables

**Table 1:** Summary of the main characteristics and the more abundant epitranscriptomic modifications in coding RNA (mRNA), and noncoding-RNAs (tRNA, rRNA, lncRNA, miRNA, circRNA).

RNA Species	Post-Transcriptional Modifications	RNA Characteristics	Ref.
mRNA	Cap 5′-end Poly-A tail 3′-end Splicing N6-methyladenosine N1-methyladenosine 5-methylcytidine Pseudouridine Adenosine to Inosine editing	Structure: The mature messenger RNA (mRNA) is a single linear polymer of ribonucleotides containing the 3′-UTR and 5′-UTR regions that border the coding sequence Function: mRNA carries the genetic information from the DNA to the ribosome where it is translated into a polymer of amino acids (protein)	[19,20,21,24,25,27,28,33]
tRNA	Anticodon loop: inosine, queuosine, 5-methylcytosine, 5-methoxycarbonylmethyl-2-thiouridine, threonyl-carbamoyl-adenosine and wybutosine D-loop: dihydrouridine T-loop: pseudouridine	Structure: The mature transfer RNA (tRNA) is a polymer of ribonucleotides with a characteristic three-dimensional structure. Here, the anticodon loop has the three pairing nucleotides with the codon in the mRNA; the 3′-end has the CCA sequence that allows the binding of the amino acid Function: The tRNA is the translator of the nucleotidic language to aminoacidic language	[19,20,28,29,91,92,99,106]
rRNA	2′-*O*-methylation Uridine-to-Pseudouridine isomerization Pseudouridine	Structure: The mature ribosomal RNA (rRNA) is a polymer of ribonucleotides consisting of several hairpin clusters Function: rRNA together with riboproteins compose the ribosome. Here, rRNAs have a direct role in the formation of the peptic bond between two amino acids	[19,20,28,127,128]
lncRNA	N6-methyladenosine N1-methyladenosine 5-methylcytidine Pseudouridine	Structure: The mature long noncoding-RNA (lncRNA) is a polymer of ribonucleotides with secondary structure Function: lncRNA has a significant role in different biological functions such as chromatin modifications, post-transcriptional regulation	[19,20,28,72,98,99,104]
miRNA	Pseudouridine 5-methylcytidine Adenosine to Inosine editing	Structure: The microRNA (miRNA) is a small oligonucleotide containing about 22 nucleotides with a single hairpin structure Function: The miRNA silencing at the post-transcriptional level of regulation of gene expression	[19,20,28,98,105,137]
circRNA	Pseudouridine N6-methyladenosine	Structure: The circleRNA (circRNA) is a polymer of ribonucleotides with circular conformation, as the 3′-end and 5′-ends have been joined together Function: The circRNA function as a sponge of miRNA, therefore have a key role in the regulation of gene expression	[19,20,28,98,138]

**Table 2:** Prediction tools of RNA modifications.

Tools	Source	Prediction of Modifications	Description	Ref.
HAMR	http://www.lisanwanglab.org/hamr/	m^1^A, m^6^A, A-to-I, Pseudouridine (Ψ), Dihydrouridine (D)	HAMR (High-throughput Annotation of Modified Ribonucleotides) is a web application that allows to detect and classify modified nucleotides in RNA-seq data	[153]
PAI	http://lin.uestc.edu.cn/server/PAI/	A-to-I	Prediction of Adenosine to Inosine sitesby using pseudo nucleotide compositions	[155]
iRNA-AI	http://lin.uestc.edu.cn/server/iRNA-AI/	A-to-I	Identification of Adenosine to Inosine editing sites	[156]
RAMPred	http://lin-group.cn/server/RAMPred/	m^1^A	Identification of the N1-methyladenosine sites in eukaryotic transcriptomes	[157]
iRNA-3typeA	http://lin-group.cn/server/iRNA-3typeA/	m^1^A, m^6^A, A-to-I	Identification of 3-types of modification at RNA’s Adenosine sites	[158]
iRNA-PseColl	http://lin.uestc.edu.cn/server/iRNA-PseColl/	m^1^A, m^6^A, m^5^C	A seamless platform for identifying the occurrence sites of different RNA modifications by incorporating collective effects of nucleotides into PseKNC	[159]
iRNAm5C-PseDNC	http://www.jci-bioinfo.cn/iRNAm5C-PseDNC/	m^5^C	Identification of RNA 5-methylcytosine sites by incorporation physical-chemical properties into pseudo dinucleotide composition	[160]
iRNA-Methyl	http://lin.uestc.edu.cn/server/iRNA-Methyl/	m^6^A	Identification of N6-methyladenosine sites using pseudo nucleotide composition	[161]
m6Apred	http://lin.uestc.edu.cn/server/m6Apred/	m^6^A	Identification and analysis of the N6-methyladenosine in Saccharomyces cerevisiae transcriptome	[162]
MethyRNA	http://lin.uestc.edu.cn/server/methyrna/	m^6^A	A sequence-based tool for the identification of N6-methyladenosine sites	[163]
SRAMP	http://www.cuilab.cn/sramp/	m^6^A	SRAMP (sequence-based RNA Adenosine methylation site predictor), a useful tool to predict m^6^A modification sites on the RNA sequences of interests	[164]
RAM-ESVM	http://server.malab.cn/RAM-ESVM/	m^6^A	Identification of M6A Sites in theS. Cerevisiae Transcriptome	[165]
PPUS	http://lyh.pkmu.cn/ppus/	Pseudouridine (Ψ)	PPUS is an online tool to predict Pseudouridine sites recognized by Pseudouridine synthase in RNA	[166]
iRNA-PseU	http://lin.uestc.edu.cn/server/iRNA-PseU/	Pseudouridine (Ψ)	Identification of RNA Pseudouridine sites	[167]
tRNAMOD	http://crdd.osdd.net/raghava/trnamod/	Pseudouridine (Ψ), Dihydrouridine (D)	Web-server for the prediction of transfer RNA (tRNA) modifications	[168]

**Table 3:** Source of the origin the diverse types of stem cells and recapitulates the main characteristics of naïve, primed, ESCs, ASCs, iPSCs, and CSCs.

Stem Cell Types	Origin	Properties	Ref.
Naïve stem cells	Zygotic stage of the mammalian embryo, immediately after the maternal redetermination. Specifically, they originate from the preimplantation epiblast	Self-renewal Pluripotency Unrestricted stem cells	[173,174,175]
Primed stem cells	Zygotic stage of the mammalian embryo, immediately after the maternal redetermination. Specifically, they originate from naïve stem cells that enter into a lineage commitment process	Self-renewal Pluripotency More lineage restricted stem cells compared to naive stem cells	[173,174,175]
Embryonic Stem cells (ESCs)	ESCs originate from the inner mass of the blastocyst	Self-renewal Pluripotent: multi-lineage differentiation through either asymmetric or symmetric division Generation of all 254 cell types originating adult tissue Generation of mouse chimeras Replacement of damaged cells with newly differentiated progenies if transplanted in degenerated tissues/organs	[170,177,196,206,207]
Adult stem cells (ASCs)	ASCs are created during ontogeny and persist in the adult tissues/organs within the niche	Self-renewal Multipotent: multi-lineage differentiation through either asymmetric or symmetric division Maintenance of the tissue homeostasis in the physiological condition Replacement of damaged cells with newly differentiated progenies if transplanted in degenerated tissues/organs	[170,178,196,206,207]
Induced pluripotentStem cells (iPSCs)	iPSCs originate in vitro from somatic differentiated cells after transduction with cMyc, Klf-4, Oct-3/4 , Sox-2 genes	Self-renewal Pluripotent Generation of patient-specific stem cells Generation of mouse chimeras Replacement of damaged cells with newly differentiated progenies if transplanted in degenerated tissues/organs	[170,179,180,196,206,207]
Cancer stem cells (CSCs)	CSCs originate from: (i) Malignant transformation of normal stem cells (ii) de-differentiation of cancer cell	Self-renewal through either asymmetric or symmetric division Multipotent: multi-lineage differentiation. Key role in predicting the biological aggressiveness of the cancer	[170,201,202,206,207]

**Table 4:** Correlation between RNA modifications and stem cells.

Stem Cell Types	RNA Modification	Presence/Absence Increase/Decrease	Effect	Ref.
Naïve vs. primed	m^6^A in mRNAs	Presence	Molecular switches for differentiation and generation of EpiSCs	[215,216]
Naïve vs. primed	m^6^A in mRNAs	Absence	Molecular switches for reating the naïve status	[215,216]
Naïve/ESCs	pseudouridylation of tRNA	Presence	Stem cell commitment during the first stage of embryogenesis	[242,243]
ESCs	m^6^A in mRNAs	Presence	Critical steps for keeping ESCs in a stemness status	[213,217,218]
ESCs	m^6^A in mRNAs	Absence	Critical steps for keeping ESCs in a stemness status	[220,221]
ESCs	m^5^C in mRNAs	Increase	Critical steps for keeping ESCs in a stemness status	[241]
ESCs	m^5^C in mt-tRNAs	Presence	Regulator of ESCs fate	[124]
ASCs	m^6^A in mRNAs	Presence	Activation of differentiation process	[224]
ASCs	m^6^A in mRNAs	Decrease	Hamper the HSCs development	[213,227,228,229]
ASCs	m^6^A in mRNAs	Decrease	Myeloid differentiation of HSCs	[230]
ASCs	m^5^C in mRNAs	Presence	Balance of epidermis stem cell self-renewal and differentiation processes	[237,238]
CSCs	m^6^A in mRNAs	Increase	Acute myeloid leukemia	[230]
CSCs	m^6^A in mRNAs	Absence/Decrease	Growth and self-renewal of human glioblastoma stem cells	[232,234]
CSCs	m^6^A in mRNAs	Decrease	Progression of human lung cancer	[235]
CSCs	m^6^A in mRNAs	Decrease	Demethylation on NANOG mRNA in breast cancer stem cells in response to hypoxia	[81]

## Abbreviations

3′-UTR	Three prime untranslated region
ALKB	Alpha-ketoglutarate-dependent dioxygenase AlkB
ALKBH1	Nucleic acid dioxygenase
ALKBH5	RNA demethylase ALKBH5
ALYREF	THO complex subunit 4
BCL2	B-cell lymphoma 2
CDS	CoDing Sequence
DKC1	H/ACA ribonucleoprotein complex subunit 4
DNA	Deoxyribonucleic acid
DNMT2	tRNA (cytosine(38)-C(5))-methyltransferase
EGFR	Epidermal growth factor receptor
ELP3	Elongator complex protein 3
FOXM1	Forkhead box protein M1
HEN1	Small RNA 2′-*O*-methyltransferase
HNRNPA2B1	Heterogeneous nuclear ribonucleoproteins A2/B1
hTRMT9	Human Probable tRNA methyltransferase 9B
IME4	N6-adenosine-methyltransferase
MED2	Mediator of RNA polymerase II transcription subunit 2
MED4	Mediator of RNA polymerase II transcription subunit 4
METTL2	Methyltransferase-like protein 2
METTL3	Methyltransferase-like protein 3
METTL6	Methyltransferase-like protein 6
METTL14	Methyltransferase-like protein 14
MYB	Myb proto-oncogene protein
MYC	Myc proto-oncogene protein
MyoD	Myogenic differentiation 1
NOTCH1	Neurogenic locus notch homolog protein 1
NSUN2	NOP2/Sun domain family, member 2
NSUN4	NOL1/NOP2/Sun domain family member 4
p16	Cyclin-dependent kinase inhibitor 2A, multiple tumor suppressor 1
PTEN	Phosphatidylinositol 3,4,5-trisphosphate 3-phosphatase and dual-specificity protein phosphatase
PUS	Pseudouridine synthase A
RBM15	Putative RNA-binding protein 15
RluE	Ribosomal large subunit Pseudouridine synthase E
ROS	Reactive oxygen specie
RPUSD3	Mitochondrial mRNA Pseudouridine synthase RPUSD3
RPUSD4	Mitochondrial RNA Pseudouridine synthase RPUSD4
SMAD2	Mothers against decapentaplegic homolog 2
SMAD3	Mothers against decapentaplegic homolog 2
SOD1	Superoxide dismutase [Cu-Zn]
SPI1	Transcription factor PU.1
SRSF2	Serine/arginine-rich splicing factor 2
TAZ	Tafazzin
TET1	methylcytosine dioxygenase TET1
TET3	methylcytosine dioxygenase TET3
TGFβ	Transforming growth factor beta
TRM4	Multisite-specific tRNA:(cytosine-C(5))-methyltransferase
TrmL	tRNA(cytidine(34)-2′-*O*)-methyltransferase
TRMT10C	tRNA methyltransferase 10 homolog C
TRMT61A	tRNA (adenine(58)-N(1))-methyltransferase catalytic subunit
TRMT61B	tRNA (adenine(58)-N(1))-methyltransferase, mitochondrial
TRUB2	Mitochondrial mRNA Pseudouridine synthase TRUB2
U1	U1 spliceosomal RNA
U2	U2 spliceosomal RNA
U3	U3 spliceosomal RNA
U4	U4 spliceosomal RNA
U5	U5 spliceosomal RNA
U6	U6 spliceosomal RNA
U8	U8 spliceosomal RNA
UHRF2	E3 ubiquitin-protein ligase UHRF2
WTAP	Wilms tumor 1 associated protein
YTDCH1	YTH domain-containing protein 1
YTHDF1	YTH domain-containing family protein 1
YTHDF2	YTH domain-containing family protein 2
YTHDF3	YTH domain-containing family protein 2
ZNF217	Zinc finger protein 217
